# Infantile Colic Treated With *Bifidobacterium longum* CECT7894 and *Pediococcus pentosaceus* CECT8330: A Randomized, Double-Blind, Placebo-Controlled Trial

**DOI:** 10.3389/fped.2021.635176

**Published:** 2021-09-10

**Authors:** Ke Chen, Changqi Liu, Hua Li, Yuehua Lei, Chenggui Zeng, Shuhong Xu, Jianqiu Li, Francesco Savino

**Affiliations:** ^1^Department of Nutrition, Chengdu Women's and Children's Central Hospital, School of Medicine, University of Electronic Science and Technology of China, Chengdu, China; ^2^Department of Child Health Care, Angel Children's Hospital, Chengdu, China; ^3^School of Exercise and Nutritional Sciences, San Diego State University, San Diego, CA, United States; ^4^Department of Child Health Care, Qingbaijiang Maternal and Child Health Hospital, Chengdu, China; ^5^Department of Child Health Care, Chengdu Caojiaxiang Community Healthcare Center, Chengdu, China; ^6^Department of Child Health Care, Huili Maternity and Child Care Center, Huili, China; ^7^Department of Paediatrics, S.S.D. Subintensive Neonatal Care, Children Hospital ‘Regina Margherita’, Turin, Italy

**Keywords:** *Bifidobacterium longum* CECT7894, *Pediococcus pentosaceus* CECT8330, infantile colic, infants, fecal consistency, probiotics

## Abstract

**Background:** Colic is a common condition in infants <4 months of age. Attempts to treat infantile colic with probiotics have shown variable efficacy and overall low evidence of success. In this work, we tested the hypothesis that oral administration of *Bifidobacterium longum* CECT7894 (KABP042) and *Pediococcus pentosaceus* CECT8330 (KABP041) mix (1 × 10^9^ colony forming units) would improve the symptoms of infantile colic.

**Methods:** A total of 112 exclusively breastfed or mixed fed infants aged <2 months and meeting the ROME IV criteria for infantile colic were recruited. The infants were randomized in a double-blind, placebo-controlled trial to receive orally administered probiotics (intervention group, IG, *n* = 48) or placebo (placebo group, PG, *n* = 42) daily for 21 days.

**Results:** Infants in the IG had significantly shorter crying time (*p* < 0.001) on day 7 [IG vs. PG, median (25−75th percentile): 38 (3.5–40.5) vs. 62 (40–108) min/day], day 14 [IG vs. PG: 20 (0–40) vs. 50 (30–75) min/day], and day 21 [IG vs. PG: 14 (0–33) vs. 40 (28–62) min/day]. Higher responder ratio and fewer crying/fussing episodes on days 7, 14, and 21 and better stool consistency on day 21 were observed in the IG (*p* < 0.01) as compared to the PG. Conversely, no significant effects on stool frequency or quality of life were observed.

**Conclusions:** In summary, daily oral administration of *B. longum* CECT7894 (KABP042) and *P. pentosaceus* CECT8330 (KABP041) was an effective treatment for shortening crying time due to infantile colic and for improving fecal consistency. This trial was registered retrospectively in December 2019 with a trial number of ISRCTN92431452.

## Introduction

Infantile colic is a behavioral condition in early infancy that involves long crying bouts and hard-to-soothe behavior for no apparent cause. This distressing condition affects up to 20% of infants under 3 months of age (1). Although infantile colic spontaneously resolves after the first 3–4 months after birth, it is associated with maternal depression, early breastfeeding cessation, and shaken baby syndrome (2). As a common problem in pediatric and family medicine practice, infantile colic imposes significant costs to healthcare sectors (3). The etiology of infantile colic remains unclear; however, various theories have been proposed such as overproduction of intestinal gas, hypersensitivity to cow's milk proteins, transient lactase deficiency, and gut inflammation (4–6). The parents and caretakers often seek medical care for colic including the use of drugs, vegetable fiber, lactase, sucrose solution, hypoallergenic diet, and herbal tea. Nevertheless, no single effective and safe intervention for infantile colic exists (7, 8).

The interest of using probiotics as a potential treatment to reduce crying in colicky infants has increased lately. Recent studies have reported that gut microbiota in colicky infants is characterized by lower proportions of *Lactobacilli* and *Bifidobacteria* and higher proportions of opportunistic proteobacteria (such as *Escherichia coli, Enterobacter aerogenes*, and *Klebsiella spp*.) (9) as compared with that in the control infants. Thus, several researchers have suggested that probiotics may be useful for treating breastfed colicky infants and reducing their crying time (10, 11).

*Pediococcus pentosaceus* CECT8330 (KABP041) can induce anti-inflammatory interleukin 10 (IL-10) production. Its combination with *Bifidobacterium longum* CECT7894 (KABP042) has shown a broad spectrum of inhibitory activities against gas-producing enteropathogens. Both strains have been isolated from fecal samples of healthy infants (12). In this randomized, double-blind, placebo-controlled trial, we aim to assess whether intervention with the two-combined probiotic strains, *B. longum* CECT7894 (KABP042) and *P. pentosaceus* CECT8330 (KABP041), as found in a currently marketed product, would reduce daily crying or fussing time (primary outcome), decrease daily episodes of crying or fussing, and improve parental quality of life (secondary outcomes).

## Materials and Methods

### Subjects and Ethical Approval

This randomized, double-blinded, placebo-controlled parallel-group study was performed in Qingbaijiang, Jinniu, and Wuhou Districts of Chengdu City and Huili County of Xichang City, Sichuan Province, China from June 1st, 2018 to June 1st, 2019. Infants diagnosed with colic by pediatricians in the outpatient care department at Qingbaijiang Maternal and Child Health Hospital, Caojiaxiang Community Healthcare Center, Huili Maternity and Child Care Center, and Angel Children's Hospital Chengdu were recruited. Nursing advices were provided to caregivers of the infants by nurses in each hospital. The enrollment and research plan were reviewed and approved by the institutional ethics committee of Angel Children's Hospital Chengdu (acting as the centralized ethics committee). Written informed consent was obtained from parents of each infant. The present study complied with the code of ethics of the World Medical Association (Declaration of Helsinki). No important changes to methodology was made after trial commencement and all planned outcomes were reported. This trial was registered retrospectively in December 2019 with a trial number of ISRCTN92431452 (http://www.isrctn.com/ISRCTN92431452).

### Inclusion, Exclusion, and Withdrawal Criteria

Infants meeting the ROME IV diagnostic criteria for infantile colic (i.e., colicky full force crying or fussing episodes lasting at least 3 h per day and occurring at least 3 days per week within 7 days prior to enrollment, confirmed with a prospectively-kept diary between baseline visit and day 1 visit) (13) were recruited. Additional inclusion criteria were: <3 months (12 weeks) of age, ≥37 weeks of gestation at birth, vaginal delivery, birth weight >2,500 g, and parents providing written informed consent. Exclusion criteria were: average weight gain <100 grams/week from birth to the last recorded weight, major medical problems (e.g., immunocompromised disease, major developmental or genetic abnormality), gastrointestinal disorder, taking antibiotics 4 weeks prior to enrollment or during the intervention, using probiotic supplements 2 weeks prior to enrollment. Participation in the study was voluntary. The parents had the right to withdraw their child from the study without providing a reason and with no loss of benefits to which the child was entitled. If parents chose to withdraw their child, the study personnel was informed immediately. The investigators had the right to terminate participation of any child at any time if they deemed it the child's best interest. Possible reasons for withdrawal of a study subject included: child's parents withdrew consent for personal reasons; child's general condition contraindicated continuing the study as judged by the study personnel or the medical expert, significant non-compliance with study protocol or lack of cooperation, serious adverse event, and loss to follow-up. To exclude organic causes of crying, infants were examined by the study pediatrician at recruitment and during the intervention.

### Determination of Sample Size

Based on a previous study (14), a sample size of 40 infants per group was estimated as sufficient to detect a significant difference in crying time between the intervention and placebo groups using a two-sided *Z* test. The significant level of the test was targeted at 0.05 with a power of 80%. Statistical power was calculated using the G*Power software (15). The sample size was rounded up 40% to account for potential attrition. As a result, a total of 112 subjects were recruited into this study.

### Study Outline

The study included five visits to the clinics: (i) a baseline visit, which corresponded to the day when parents were referred to the pediatrician with the main complaint of the baby's crying and/or fussing without a definite reason; (ii) day 1 visit, when the ROME IV colic diagnostic criteria were verified and when participants were randomized for intervention; and (iii) day 7, 14, and 21 visits, that corresponded to the weekly assessments during the 3-week intervention phase from day 1. Caregivers were reminded of the study instructions on each visit.

### Randomization and Intervention

A completely randomized schedule that maintains balance between treatment arms in a 1:1 proportion was prepared by an independent statistician, who was not directly involved in the analysis of the study results. The RAND function of Excel (Microsoft, Redmond, WA, USA) was used to generate randomly permutated codes. The physicians responsible for enrollment of patients allocated the next available number to the subject. At enrollment, the parents of the patient received a closed envelope containing a written usage guidance for the oil drops. To minimize potential biases, the study was double-blinded whereby treatment allocation was concealed from all study investigators and participants. Drug containers prepared by an independent pharmacy were sequentially numbered and opened sequentially. Containers were of identical appearance, tamper-proof, and equal in weight. All the caregivers have been well-trained by using a standardized protocol.

All eligible colicky infants were randomized to receive either the probiotics (intervention group, IG) or a reference product without the probiotics (placebo group, PG). There was no delay between randomization and the initiation of the intervention. The probiotic formula comprised a pure sunflower oil suspension (not an emulsion) of strains *B. longum* CECT7894 (KABP042) and *P. pentosaceus* CECT8330 (KABP041). Each five drops of the formula contained one billion total colony-forming units (CFU) in a 1:1 proportion for the two strains, as stated by the manufacturer under ICH zone-II stability conditions. However, the exact counts in the clinical batch were not independently verified in this study. The reference product (placebo) contained an identical sunflower oil, without the probiotics. Both the probiotic and the placebo were manufactured by PROCEMSA srl (Italy) for AB-Biotics SA (Spain), the company owning IP rights of the strains. PROCEMSA has GMP and ISO9001 certifications and tests the quality of each batch using the European pharmacopeia-compliant methods for total yeasts and molds, total Gram-negative bile-tolerant bacteria, *E. coli, Salmonella, Staphylococcus aureus, Listeria monocytogenes*, and peroxides. Additionally, PROCEMSA tests for heavy metals using ICP-OES. Products are routinely shipped from Spain to China in containers controlled for temperature with data loggers. Shelf life of the product is 2 years from manufacturing when stored at room temperature (25°C) or below. Expiry date is noted on boxes. The products were used before reaching the expiry date.

Caregivers, who received the standardized operation training from nurses at the time of recruitment, administered five drops of the study product orally to each infant, daily for 21 days. Caregivers were instructed to give five more drops if regurgitation occurred right after the first administration. The dose was not required to be given at a fixed time or given with feeds. However, for compliance and ease of administration, each family has been recommended to give the dose with the same feed each day. When the administration was completed, the empty bottle was retrieved to assess the adherence.

### Blinding

The probiotics and the placebo products were indistinguishable in smell, taste, and appearance. Both products were only labeled with the randomization number, batch number, expiry date, and the statement “For clinical trial use only.” The random allocation sequence was generated by an independent statistician. Caregivers of each participant received a numbered treatment bottle based on the sequence. Parents of the subjects, the clinical team, statisticians, and representatives from AB-Biotics S.A were blinded during the entire study until the database was unlocked.

### Outcome Measures

The primary outcome (daily crying or fussing time) and secondary outcome (number of crying/fussing episodes, fecal consistency, and parental quality of life) assessments were adapted from Sung et al. with minor modifications (16) and were measured in all study visits. The proportion of responders [defined as subjects who had a reduction in colicky full force crying/fussing time of ≥50% from baseline according to a previous publication (10)] were calculated on the 7th, 14th, and 21st days of the intervention. The validated Barr diary (17) was used to record the infant colicky full force crying/fussing time (mins/day), number of episodes of colicky full force crying/fussing/day, stool consistency, and stool frequency. Stool consistency on diapers was scored as 0 for watery stool, 1 for loose stool, 2 for formed stool, and 3 for hard stool as per Amsterdam consistency subscale (18). PedsQL^TM^, a 15-item validated questionnaire, was used to assess family functioning (19), each item being scored based on its frequency (0: never happened, 1: almost never happened, 2: happened sometimes, 3: happened often, 4: almost always happened, and 5: always happened). The nurses explained to the children's caregivers how to properly record all the information. There were no changes to trial outcome measures after the trial commenced. In addition, demographic information was collected by a questionnaire at baseline. Potential confounders were also recorded at baseline (family history of atopy, antenatal or current probiotic/antibiotic use, and smoking during pregnancy) and during the intervention [infant feeding method (breast vs. formula); mother's intake of dairy products, probiotics, and medications; infant's intake of dairy products, probiotics, solids, and medications; concurrent illnesses/immunizations]. Compliance was measured by the number of days over the preceding week when the study drops were not administered. Occurrence of side effects was monitored throughout the study.

### Statistical Methods

The distribution of the data was tested for normality prior to analysis using the Shapiro-Wilks normality test. Baseline characteristics and study outcomes were presented as mean and standard deviation (SD) for normally distributed variables or median and 25th and 75th percentiles (P25, P75) for variables with a skewed distribution. All tests of significance were two-tailed with an α = 0.05. The Student's *t* test was used to compare the mean values of continuous variables approximating a normal distribution. For non-normally distributed variables, the Kruskal-Wallis or chi-square test was used. Relative risk based on the proportion of responders on the 7th, 14th, and 21st days during the intervention and its 95% confidence interval were calculated. Data were analyzed using the SAS for Windows statistical software package (SAS Institute Inc., Cary, NC, USA).

## Results

### Participant Characteristics

**Figure 1:**
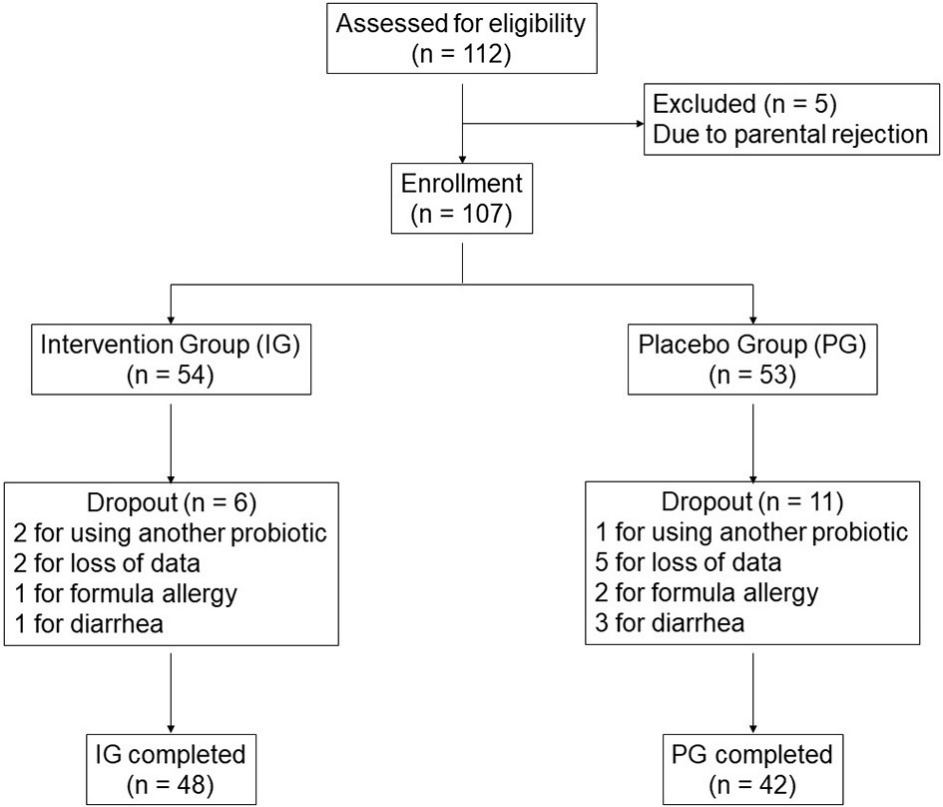
Diagram of patient enrollment and study progress.

A total of 112 infants met the inclusion criteria (56 for each group). Five infants were excluded due to parental rejection. Of the remaining 107 infants, 17 (15.9%) dropped out during the study. Three of these infants were withdrawn for using other probiotics, seven for loss to follow-up, three for formula-related allergy (formulas used in the study were of the partially hydrolyzed type), and four for diarrhea. Of note, antacid medication is normally not used in colicky infants in China. No infant in the study was taking antacid medication. Thus, the primary and secondary outcome measures were obtained from 90 infants (48 for the IG and 42 for the PG) (Figure 1). Despite the drop-outs, the study sample size (*n* = 48 for the IG and *n* = 42 for the PG) retained 80.9% power for crying time, 99.1% power for number of episodes, and 81.6% power for responder rate on day 21, when the differences between the IG and the PG were the smallest during the 7–21 day period. Even higher statistical powers were reached on days 7 and 14.

There were no significant differences in gender, allergy history, age, birth weight, passive smoking exposure, living environment, parents' educational levels (all above illiteracy, primary school, and middle school levels), and feeding method during the intervention between the two groups (*p* > 0.05) (Table 1). Most infants were exclusively breastfed during the study period, few had mixed feeding (all of them being >50% breastfed), and none received water-based products or fruit juices. No treatment-related side effects occurred during the study period.

**Table 1:** Clinical and socioeconomic data of the infants.

**Index**	**IG^a^**	**PG^b^**	***p*-values**
No.	48	42	-
Female [*n* (%)]^c^	22 (45.8)	21 (50.0)	0.693
Gestational age (weeks) [mean ± SD^d^]^e^	39.7 ± 4.9	38.4 ± 4.4	0.904
Age when recruited (weeks) [mean ± SD]^e^	0.99 ± 0.4	0.81 ± 0.5	0.969
Birth weight (kg) [mean ± SD]^e^	3.41 ± 0.7	3.53 ± 0.7	0.204
No. of probiotics use in pregnancy [*n* (%)]^c^	15 (31.3)	11 (26.2)	0.597
No. of infants with allergic history [*n* (%)]^c^	2 (4.2)	3 (7.1)	0.539
No. of passive smoking exposure [*n* (%)]^c^	0 (0.0)	0 (0.0)	1.000
Living environment [*n* (%)]^c^			0.465
Rural	7 (14.6)	4 (9.5)	
Urban	41 (85.4)	38 (90.5)	
Levels of parents' education [*n* (%)]^c^			0.196
High school	2 (4.2)	0 (0)	
Junior college	4 (8.4)	2 (4.8)	
University	42 (87.4)	40 (95.2)	
Feeding method during intervention [*n* (%)]^c^			0.257
Exclusively breastfed	38 (79.2)	37 (88.1)	
Mixed fed^f^	10 (20.8)	5 (11.9)	

a *IG, intervention group;*

b *PG, placebo group;*

c *chi-square test for comparison between groups;*

d *SD, standard deviation;*

e *t-test for comparison between groups;*

f *all being > 50% breastfed*.

### Effect of Intervention on Primary Outcome: Crying/Fussing Time

**Figure 2:**
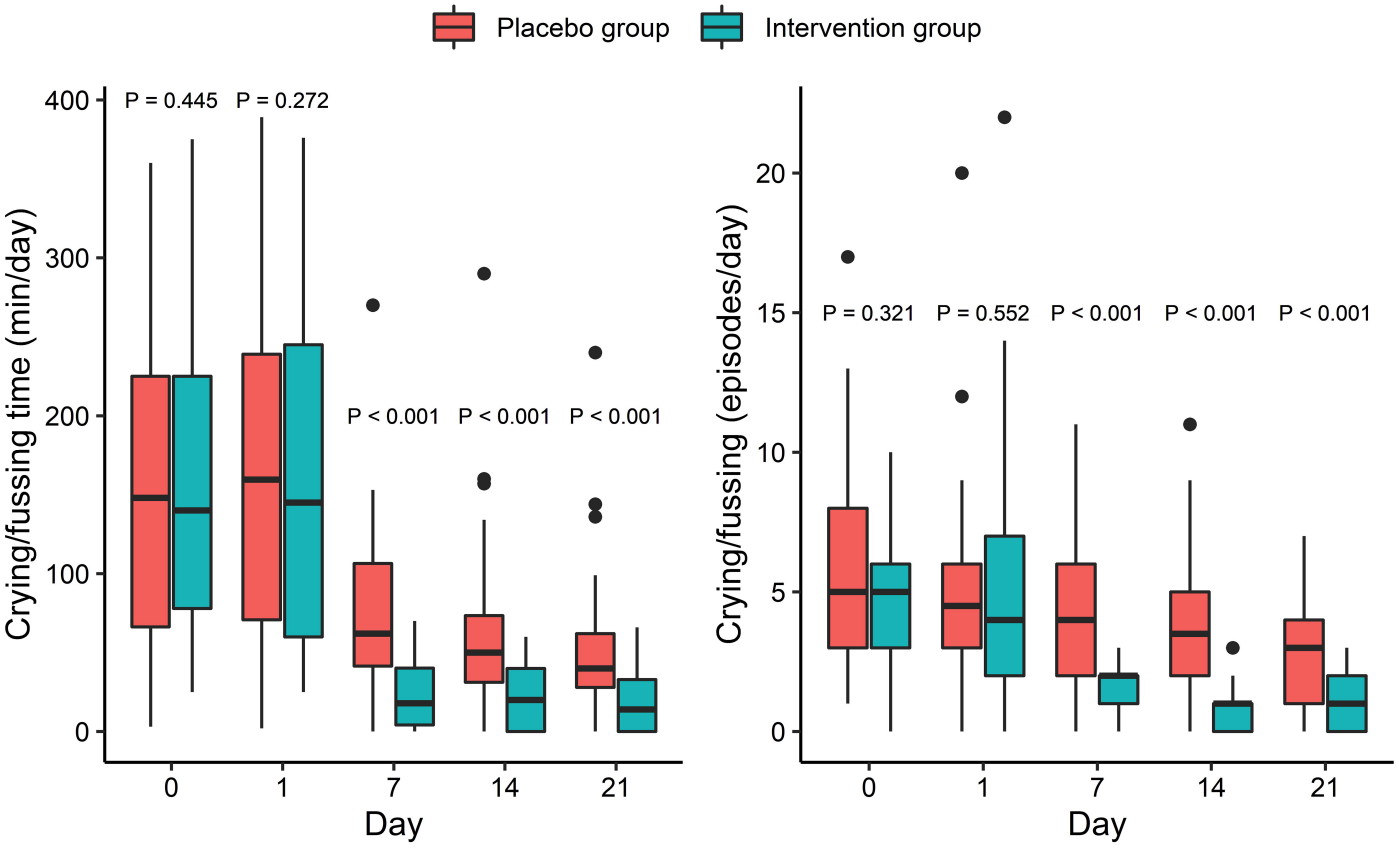
Effect of intervention on crying/fussing time (left) and number of crying/fussing episodes (right). P-values correspond to Kruskal-Wallis test performed at each timepoint. Box and whiskers denote quartiles and 95% percentiles.

The Shapiro-Wilks normality test showed that crying/fussing time and frequency were both non-normally distributed variables. Therefore, the Kruskal-Wallis test was used to compare the difference between the two groups at different time points. There were no significant differences in crying/fussing time and frequency between the IG and the PG at baseline (*p* > 0.05, Figure 2). However, crying/fussing time in the IG was significantly shorter than that in the PG on the 7th, 14th, and 21st days of the intervention (*p* < 0.001, Figure 2).

### Effect of Intervention on Secondary Outcomes: Crying/Fussing Frequency, Fecal Frequency, Fecal Consistency, and PedsQL™ Family Impact Subscales

The crying/fussing frequency, fecal frequency, and fecal consistency scores were all non-normally distributed variables and thus the Kruskal-Wallis test was used to assess the differences between the groups. There were no significant differences in crying/fussing frequency between the IG and the PG at baseline (*p* > 0.05, Figure 2). Nevertheless, the frequency of the crying/fussing episodes in the IG was lower than that in the PG on the 7th, 14th, and 21st days of the intervention (*p* < 0.001, Figure 2). Also, a higher proportion of responders was observed in the IG as compared with the PG on the 7th (83.3 vs. 35.7%, *p* < 0.001), 14th (95.8 vs. 42.9%, *p* < 0.001), and 21st (89.5 vs. 64.3%, *p* = 0.004) days (Table 2).

**Table 2:** Number of responders to the treatment.

**Index**	**IG^a^ (*n* = 48)**	**PG^b^ (*n* = 42)**	**RR^c^ (95% CI)**	***p*-values**
Day 7 [*n* (%)]	40 (83.3)	15 (35.7)	2.333 (1.695–5.973)	<0.001
Day 14 [*n* (%)]	46 (95.8)	18 (42.9)	2.236 (1.569–3.186)	<0.001
Day 21 [*n* (%)]	43 (89.5)	27 (64.3)	1.394 (1.090–1.781)	0.004

a *IG, intervention group;*

b *PG, placebo group;*

c *RR, relative risk*.

As shown in Table 3, there were no significant differences in fecal frequency and consistency score between the IG and the PG before the intervention (*p* > 0.05). Infants in the IG had lower fecal frequency/day than infants in the PG. The differences were already significant on the 1st day and further increased on the 7th, 14th, and 21st days (*p* < 0.001, Table 3). Conversely, the fecal consistency score of infants in the IG was significantly higher than that of infants in the PG on the 21st day of intervention only (*p* < 0.001, Table 3).

**Table 3:** Effect of intervention on fecal consistency and frequency.

**Index**	**IG^a^ (** ***n*** **= 48)**		**PG^b^ (** ***n*** **= 42)**		**Chi-square test** ***p*-values**
	**Median** ** (25−75th percentile)**	**Mean ± SD^c^**	**Median** ** (25−75th percentile)**	**Mean ± SD**	
**Fecal frequency/day**					
Baseline	4 (3–5)	4.00 ± 1.74	4 (3–6)	4.50 ± 1.86	0.337
Day 1	3 (2–4)	3.10 ± 1.70	4 (3–5)	4.24 ± 1.79	0.004
Day 7	2 (1.5–3)	2.35 ± 1.34	4 (3–5)	4.02 ± 1.66	<0.001
Day 14	2 (1–3)	2.25 ± 1.33	3 (3–4)	3.55 ± 1.77	<0.001
Day 21	2 (1–2)	1.84 ± 0.98	3 (2–4)	3.18 ± 1.39	<0.001
**Fecal consistency score**					
Baseline	0.85 (0.65–1.00)	0.83 ± 0.40	0.96 (0.74–1.00)	0.85 ± 0.22	0.337
Day 1	0.90 (0.50–1.00)	0.77 ± 0.37	0.92 (0.67–1.03)	0.85 ± 0.27	0.757
Day 7	1.00 (0.78–1.20)	1.06 ± 0.61	1.00 (0.81–1.14)	0.95 ± 0.36	0.846
Day 14	1.00 (0.53–1.00)	0.86 ± 0.45	1.00 (0.75–1.19)	1.07 ± 0.25	0.471
Day 21	1.00 (0.99–1.27)	1.02 ± 0.39	1.00 (0.62–1.02)	0.85 ± 0.41	<0.001

a *IG, intervention group;*

b *PG, placebo group;*

c *SD, standard deviation*.

There were no significant differences in the PedsQL™ Family Impact Subscales between the IG and the PG at baseline (*p* > 0.05, Table 4). The Physical Function Subscale in the IG was marginally improved without reaching significance when compared with infants in the PG on the 21st day (*p* = 0.065) and a similar result was found for the Social Function Subscale on the 14th day (*p* = 0.070, Table 4). Finally, no significant side effects occurred during the study period.

**Table 4:** Effect of intervention on PedsQL™ family impact subscales.

**Index**	**IG^a^ (** ***n*** **= 48)**		**PG^b^ (** ***n*** **= 42)**		***p-values***
	**Median (25−75th percentile)**	**Mean ± SD^c^**	**Median (25−75th percentile)**	**Mean ± SD**	
**Physical functional score**					
Baseline	6 (1–9)	6.35 ± 5.67	8 (5–11)	7.62 ± 4.82	0.140
Day 7	6 (2–10)	6.58 ± 5.69	5 (2–8)	5.57 ± 3.93	0.533
Day 14	6 (0.5–10)	5.96 ± 4.92	6 (3–8)	5.52 ± 3.52	0.754
Day 21	6 (1–9)	5.56 ± 4.29	4 (2–6)	3.79 ± 2.53	0.065
**Emotional functional score**					
Baseline	4.7 (0–7.0)	5.50 ± 3.90	4.74 (2–7)	4.50 ± 3.77	0.971
Day 7	3.5 (1–5.0)	3.31 ± 2.43	3.0 (0–6.5)	4.13 ± 4.60	0.873
Day 14	5 (0–8)	5.52 ± 6.11	3.5 (0–6)	3.69 ± 3.64	0.163
Day 21	1 (0–5)	2.44 ± 2.66	4 (0–6)	3.80 ± 3.56	0.115
**Social functional score**					
Baseline	4 (0.5–7)	4.25 ± 3.53	4 (1–7)	4.50 ± 3.54	0.744
Day 7	3 (0–6.5)	3.81 ± 3.94	5 (2–8)	5.02 ± 4.21	0.154
Day 14	2 (0–6)	3.60 ± 3.84	4.5 (1–8)	4.79 ± 3.57	0.070
Day 21	2 (0–6)	3.00 ± 3.35	4 (0–4)	3.05 ± 2.32	0.614

a *IG, intervention group;*

b *PG, placebo group;*

c *SD, standard deviation*.

## Discussion

Administration of *B. longum* CECT7894 (KABP042) and *P. pentosaceus* CECT8330 (KABP041) at a dose of 10^9^ CFU per day to exclusively breastfed or mixed fed infants was superior to the placebo for the management of infantile colic. The use of the two strains significantly reduced the crying/fussing time and the frequency of episodes. No differences were observed on stool consistency until day 21, when a small but significant increase in consistency was observed in the IG group. Conversely, differences in the stool frequency were already apparent on day 1 and became more significant during the study. However, the fact those differences were already present on day 1 precludes attributing the effect to the probiotic intervention. Besides, no significant effects were observed on family quality of life. No adverse events and unintended effects were recorded during the intervention. *Pediococcus* strains have been used as probiotics in other clinical trials to treat several diseases, including obesity (20), diarrhea (21), trauma (22), and *Helicobacter pylori* infection (23). Strains belonging to *Lactobacillus* and *Bifidobacterium* groups have been previously trialed in colicky infants, while evidence for *Pediococcus* is scant. However, one must consider that *Pediococcus* are not only phylogenetically close to the *Lactobacillus* genus (24), but are also found in breast milk (25).

Our results were in agreement with previous studies that used probiotics to treat infantile colic. The administration of *Lactobacillus reuteri* DSM 17938 improved colic symptoms, although the effectiveness has only been seen in breastfed infants and not in formula-fed infants (11, 26). Another study showed that treatment with a combination of *L. casei, L. rhamnosus, Streptococcus thermophilus, B. breve, L. acidophilus, B. infantis, L. bulgaricus*, and fructooligosaccharides (FOS) reduced the duration of crying by almost 35 min compared to the placebo (27). Moreover, Saavedra et al. and Ivakhnenko et al. reported reduced incidence of caregiver-reported colic when infants were supplemented with a combination of *B. animalis* subsp. *lactis* BB-12 and an unidentified *S. thermophilus* strain, although colic was not formally diagnosed by a physician which reduced the strength of the studies (28, 29). A recent study on the same BB-12 strain overcame this shortcoming by formally diagnosing colic using the Rome-III criteria (30). However, this study showed that the response rate was not significantly improved against placebo until day 21, while in our study a significant improvement was observed from day 7. Conversely, a study reported that the use of *L. rhamnosus* GG (ATCC53103) had no significant effect on crying of colicky infants (31). In another study, no significant differences in crying and irritability were found between the probiotics and placebo groups when supplemented with either *L. reuteri* ATCC55730 or *B. lactis* BB-12 (32).

Possible placebo effect should be recognized (33). Previous studies have shown that placebo response rates in trials on infantile colic could range from 5 to 83% (10, 34, 35). In the present study, 27 of 42 (64.3%) infants responded to the placebo at day 21. Moreover, very short crying times were reported toward the end of the study in both probiotic and control groups, further indicating the existence of a placebo effect. Although a direct placebo effect in young infants is unlikely, an indirect placebo effect (for example, the different degree of tolerance, attention and/or care skills of caregivers to the crying infant, or subjectivity in the recall of exact crying time) may be possible. Another factor contributing to the placebo effect is the natural regression to mean (subjects are enrolled when most symptomatic and inevitably improve with time owing to the natural variation in symptom severity and irrespective of trial participation) (36, 37).

The exact mechanisms by which *B. longum* CECT7894 (KABP042) and *P. pentosaceus* CECT8330 (KABP041) might exert this action have yet to be elucidated. Intestinal inflammation has been discussed as one of the possible causes of infantile colic, together with dysbiosis, production of gas (such as H_2_), and hypersensitivity to some nutrients (4–6). In this regard, *P. pentosaceus* CECT8330 has been reported to induce the anti-inflammatory cytokine IL-10 (12, 38). In addition, *P. pentosaceus* CECT8330 (KABP041) and especially *B. longum* CECT7894 (KABP042) were able to inhibit the growth of a wide spectrum of opportunistic gas-producing enterobacteria of the *Escherichia* and *Klebsiella* genera, known to be abnormally abundant in colicky infants (39, 40). This is consistent with the reported capacity of *Bifidobacterium* strains to modulate the intestinal microbiota (41) and the antimicrobial activity of *P. pentosaceus* strains (42, 43). Additionally, *B. longum* CECT7894 (KABP042) is effective in inhibiting the growth of *Ent. aerogenes*. Future studies should aim at elucidating this effect by exploring microbiome, metabolome, and inflammation markers (e.g., fecal calprotectin).

## Strength and Limitation Analysis

The strengths of our study include adequate sample size for the predefined outcomes, proper blinding maintained throughout the treatment, data management, and analyses. In addition, high retention and reported adherence rates allowed the achievement of the predetermined statistical power and significance. Moreover, a generally accepted definition of colic was used for diagnosis (ROME-IV criteria), and infants were recruited at a similar, early age. Similar to previous studies mentioned above, a potential limitation of this study is the assessment of the duration and frequency of colicky full force crying and fussing in infants with colic relied solely on the caregivers' report. Thus, we used a placebo-controlled, blinded design to minimize this potential shortcoming. Moreover, the design of our study did not allow for a better description of the crying (e.g., no difference for the food-related crying and typical colicky full force afternoon crying). Another limitation was that only a non-significant effect could be observed in parental quality of life despite a significant effect on crying/fussing. This could be due to an inadequate sample size for this outcome or other factors having a stronger effect on parental quality of life than the baby's crying (e.g., impact on jobs or family pressure). Another limitation of this study is that the compliance with the study products was not objectively assessed. A potential approach to assessing compliance is to weigh the study bottles both before and after dispensing; however, this method has reportedly produced highly variable results (44). Finally, although sufficient for the desired statistical power, the sample size used in this study is too small to derive a definitive conclusion about the universal usefulness of this probiotic formula for teating infantile colic. Therefore, this study should be replicated with larger sample sizes, and preferably with a sufficient representation of all feeding types.

## Conclusions

In summary, exclusively breastfed or mixed fed infants with colic benefited from the treatment with *B. longum* CECT7894 (KABP042) and *P. pentosaceus* CECT8330 (KABP041) mix in comparison to the placebo. We recommend these probiotics for reducing crying times of colicky infants. Future studies should clarify the mechanism of the mix of probiotics in the management of infantile colic.

## Data Availability Statement

The raw data supporting the conclusions of this article will be made available by the authors, without undue reservation.

## Ethics Statement

The studies involving human participants were reviewed and approved by Institutional ethics committee of Angel Children's Hospital Chengdu. Written informed consent to participate in this study was provided by the participants' legal guardian/next of kin.

## Author Contributions

KC and FS designed the research. KC, HL, YL, CZ, SX, and JL were responsible for the collection and assembly of the data. KC and CL analyzed the data. KC, FS, and CL wrote the first draft of the manuscript. All authors critically reviewed the manuscript, contributed to its revision, and approved the final manuscript.

## Funding

This work was supported by the Maternal and Infant Health and Care Science Laboratory, Shanghai, China (MIHC/2017/10/AKO2018).

## Conflict of Interest

The authors declare that the research was conducted in the absence of any commercial or financial relationships that could be construed as a potential conflict of interest. AB-Biotics SA (Barcelona, Spain) is the company owning worldwide IP rights of the probiotic strains used in this research and PROCEMSA Spa (Nichelino, Italy) is a toll manufacturer employed by AB-BIOTICS SA. These two companies did not participate in study funding, design, data collection, analysis, and interpretation, or in writing the manuscript. Maternal and Infant Health and Care Science Laboratory (Shanghai, China) holds an interest in the distribution of the probiotic used in this study in China, but its contribution was limited to funding the study and publication, and did not participate in study design, data collection, analysis, and interpretation, or in writing the manuscript. The authors are not employed by any of the aforementioned companies nor hold their shares. The companies did not compensate for time and efforts of the authors in cash or kind for conducting this study.

## Publisher's Note

All claims expressed in this article are solely those of the authors and do not necessarily represent those of their affiliated organizations, or those of the publisher, the editors and the reviewers. Any product that may be evaluated in this article, or claim that may be made by its manufacturer, is not guaranteed or endorsed by the publisher.
